# A tree shrew glioblastoma model recapitulates features of human glioblastoma

**DOI:** 10.18632/oncotarget.15225

**Published:** 2017-02-09

**Authors:** Yaohui Tong, Junjun Hao, Qiu Tu, Hualin Yu, Lanzhen Yan, Yuan Li, Longbao Lv, Fei Wang, Antonio Iavarone, Xudong Zhao

**Affiliations:** ^1^ School of Life Sciences, University of Science and Technology of China, Hefei, Anhui 230026, China; ^2^ Key Laboratory of Animal Models and Human Disease Mechanisms of Chinese Academy of Sciences, Key Laboratory of Bioactive Peptides of Yunnan Province, Kunming Institute of Zoology, Kunming 650223, Yunnan, China; ^3^ State Key Lab of Genetic Resources and Evolution, Kunming Institute of Zoology, Chinese Academy of Sciences, Kunming, Yunnan 650223, China; ^4^ Kunming College of Life Science, University of Chinese Academy of Sciences, Kunming, Yunnan 650204, China; ^5^ Department of Neurological Surgery, The First Affiliated Hospital, Kunming Medical University, Kunming, Yunnan, 650032, China; ^6^ Kunming Primate Research Center, Chinese Academy of Sciences, Kunming, Yunnan 650223, China; ^7^ Kunming University of Science and Technology, Kunming, Yunnan, 650500, China; ^8^ Institute for Cancer Genetics, Columbia University, New York, New York 10032, USA

**Keywords:** glioblastoma, animal model, tree shrew, P53

## Abstract

Tupaia belangeri (tree shrew), an animal species whose genome has significantly higher similarity to primates than rodents, has been used in biomedical research. To generate animal models that reproduce the human tumors more faithfully than rodents, we present the first report of a cancer model mimicking human tumor genetics in tree shrew. By engineering a lentiviral system for the transduction of mutant H-Ras and a shRNA against tree shrew p53, we successfully generated malignant glioma in tree shrew. The tree shrew glioma exhibited aggressive behavior and a relatively short latency, and markedly reduced animal survival. Remarkably, the biological features of human high-grade glioma (necrosis, microvascular proliferation, pseudopalisading) were all present in tree shrew glioma. Furthermore, genetic analysis of tree shrew glioma revealed that the tumors were clustered within the mesenchymal subgroup of human glioblastoma multiforme. Compared with the corresponding mouse glioma, tree shrew gliomas were markedly more similar to human glioblastoma at gene expression profile. The tree shrew glioma model provides colleagues working in the field of gliomas and cancer in general with a more accurate animal model.

## INTRODUCTION

Glioblastoma, the most frequent and lethal glioma, accounts for up to half of malignant brain tumors [1]. It is one of the most lethal tumor, with a five-year survival rate of less than 5%. Survival times of GBM patients haven't been considerably prolonged despite the development of various types of advanced early diagnosis and therapies that have significantly improved the survival rates of many other cancers. The current first-line treatment for glioblastoma is surgery, followed by radiotherapy and temozolomide; however, the average survival time of glioblastoma patients is 15 months [2, 3]. Due to the emerging demands for exploring novel therapy on glioblastoma, TCGA (The Cancer Genome Atlas) project led by the National Institutes of Health, listed glioblastoma as one of three pilot studies of cancer genomics.

Rodent models of cancer have played essential role in cancer research and development of novel therapies. Currently, several types of animal model are widely used in research and therapy development. Xenograft models, in which established human cancer cell lines or patients-derived cancerous tissue are usually implanted into immunocompromised mice, cannot mimic the relationships between the immune system and developing cancer. An alternative choice is provided by syngeneic xenograft models in which mouse cancer cells are injected into the inbred strain from which the donor cells were derived. Although the immune environment keeps intact in syngeneic xenograft model, this model also cannot recapitulate the transformation process of normal cells. Genetically engineered mouse models (GEMM) have become more popular in research and drug discovery due to their advantages in fine control of genetic alterations driving human cancer development and in the normal micro-environment where the whole process of cancer development, including initiation, progression, maintenance and metastasis, is in the context of normal immune system [4]. The cancer model induced by locally transformation of adult somatic cell usually via viral infection, such as retroviral and lentiviral, has further advantage over germline engineering models in mimicking the clonal origin of human cancer and the timing of genetic alterations which most of them are gained in human cancers during adulthood.

There are numbers of GEMM models of glioblastoma that are involved in EGFRVIII, PTEN, Ink4a/Arf, TP53, NF-1, PDGF etc, which are mutated frequently in human glioblastoma. Somatic lentiviral infection overexpressing constitutively active Ras and AKT in the brains of wild type or p53+/− mice generates glioblastoma [5]. Our recent study showed that H-Ras overexpression and p53 silencing via lentiviral infection efficiently induced high-grade glioma, which was identified as mesenchymal glioblastoma, the most malignant subgroup of glioblastoma, by transcriptome analysis [6]. These findings suggest that AKT activation may not be necessary for glioma induction by Ras activation and p53 inactivation.

However, rodents have its congenital shortcomings. Only 85% of human genes have the homologous orthologues in mouse, and more than 20% of orthologues have significantly different functions according to the incomplete statistics [7]. For cancer research, human cancer cells in xenograft models locate in a microenvironment quite different from human cancer, especially the absence of intact immune system in addition to species difference between human and mouse, and it cannot mimic the transformation process of normal cells. Although the GEMM models possess intact immune system, which is very different from human immune system in structure, innate and adaptive immunity, such as mice have significant bronchus-associated lymphoid tissue, this is largely absent in healthy humans, and paneth cell defensins, and Toll-like family of receptors, and the balance of lymphocytes and neutrophils in adults, and development and regulation of B cells, T cells and NK cells [8, 9]. These pitfalls of mouse model make 90% of drug candidates successful in preclinical mouse model fail to be approved for human use.

Tree shrew is a small primate-like animal which is evolutionally closest to primate at whole genomic level [10]. It is more and more used in biomedical research to replace primate in some cases [11]. Tree shrew brain is well developed with structures resembling primates. All 23 known neurotransmitter transporters and most of visually related human genes are detected in tree shrew. It suggests tree shrew may be a good model organism for brain research.

In this study, we established a glioblastoma model in the tree shrew by intracranial injection of a lentivirus that overexpresses constitutively active H-Ras and silences the Tp53 gene. Histological and transcriptome analysis demonstrated that the glioblastoma belongs to the mesenchymal subtype and shows more similarities to human glioblastoma than mouse models induced through the same type of genetic engineering.

## RESULTS

### Comparison of human, mouse and tree shrew p53 proteins

**Figure 1:**
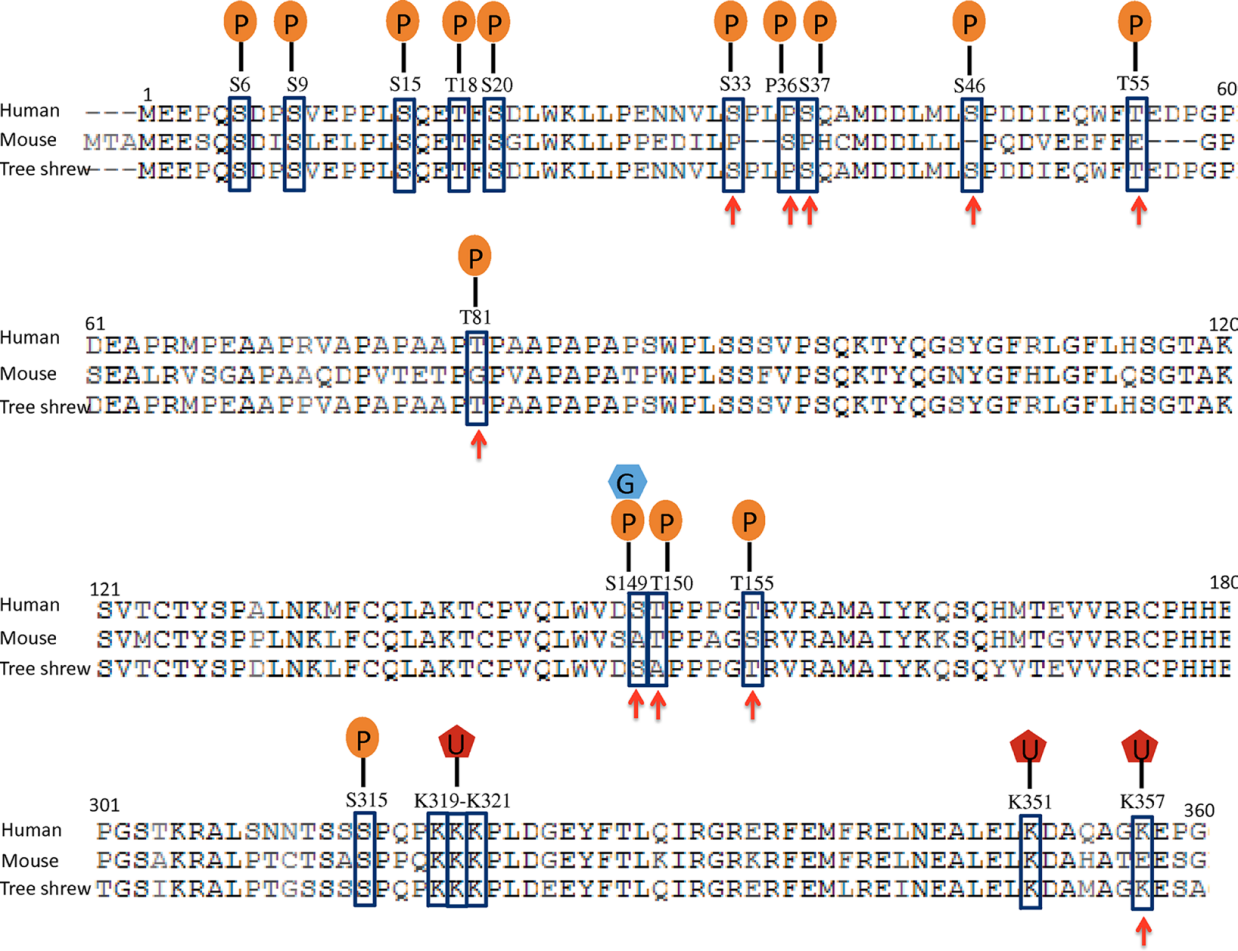
Comparison of human, tree shrew and mouse Tp53 proteins Post-translational modification sites are indicated with squares, and the sites that are conserved in tree shrew but not in mouse are indicated by arrowheads. P: phosphorylation site; G, O-GlcNAcylation; U, mono-ubiquitination.

*Tp53* mutations are found in more than 50% of all human cancers, and *Tp53* is one of the most frequently mutated genes in glioblastoma. The combination of Ras overexpression and *Tp53* silencing in hippocampal cells efficiently induces mesenchymal glioblastoma in mice. We compared the sequence homology and post-translational modifications of human, mouse and tree shrew TP53 proteins. The human and mouse TP53 proteins share only 77% sequence homology, while the tree shrew TP53 proteins shared 93% sequence homology with human. More importantly, the tree shrew TP53 protein is conserved at all phosphorylation sites except T150, but the mouse TP53 protein lacks the phosphorylation sites at S33, S36, S37, S46, T55, T81, S149 (which is also a site for O-GlcNAcylation) and T155 (Figure 1). Among these phosphorylation sites, phosphorylation at S149, T150 or 155 results in p53 degradation via the COP9 (constitutive photomorphogenesis 9) pathway; phosphorylation at S33, S37, S46, T81 or S149 stabilizes the p53 protein by blocking proteasome-dependent degradation; phosphorylation at S33, S37, S46 or T81 releases TP53 from a repression state; T55 phosphorylation results in exportation of the p53 protein from the nucleus to the cytoplasm; and S46 phosphorylation can enhance promoter-specific DNA binding of the p53 protein [12–14]. Mono-ubiquitination at K357, which leads to TP53 protein nuclear export [15], is also conserved in tree shrew but not in mouse. These post-translational modification sites, enzymes, and their association with cancer are summarized in Supplementary Table 1. In addition, while tree shrew is evolutionally closer to mouse at whole genomic level, its TP53 protein is in a cluster together with the primates (Supplementary Figure 1). These differences suggest that modeling *Tp53*-driven cancer in tree shrew may better recapitulate human disease than mouse models.

### Glioblastoma induction in tree shrew

**Figure 2:**
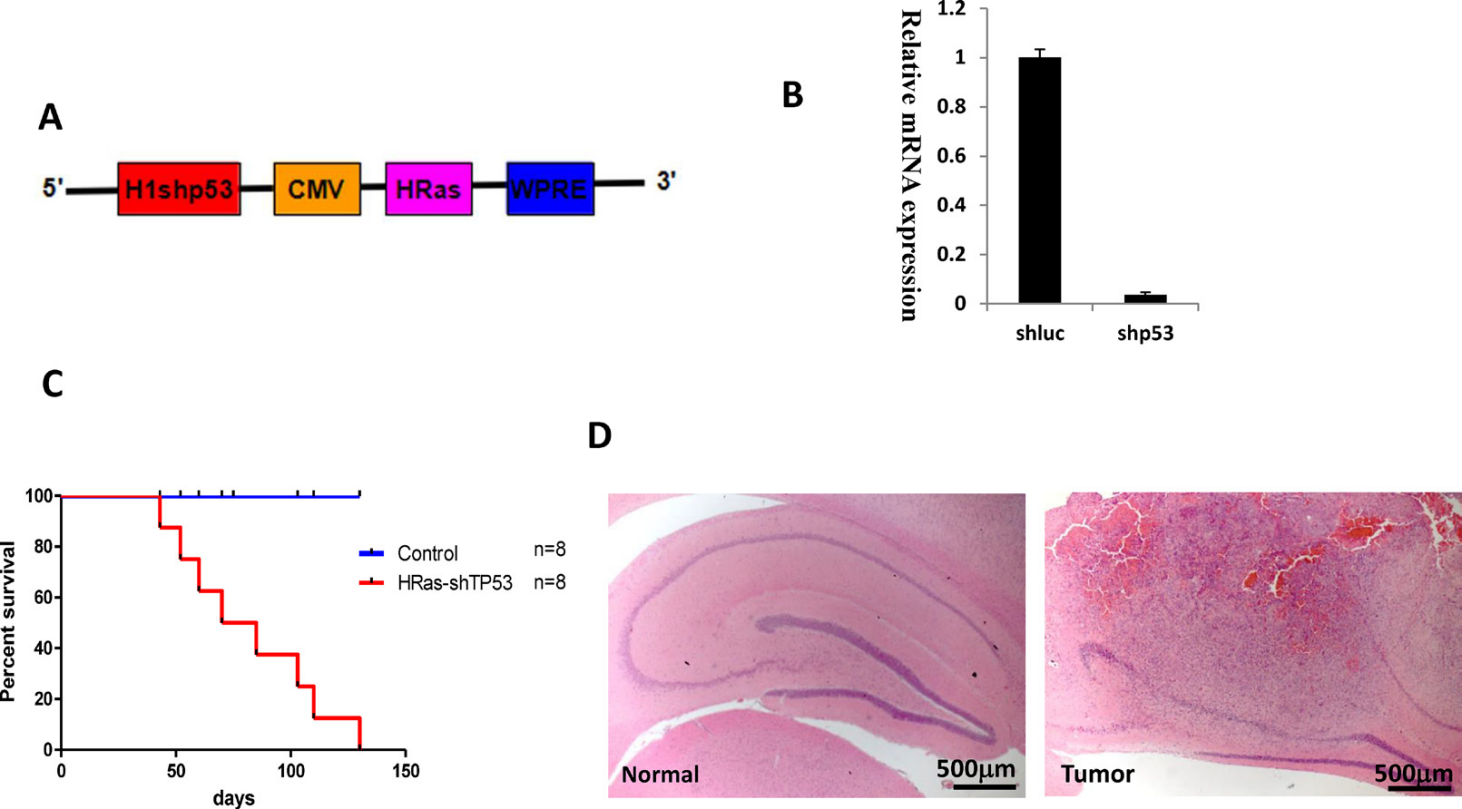
Brain tumors in tree shrew induced by overexpression of constitutive H-Ras and tp53 silencing (shp53) (**A**) Diagram of the lentiviral vector pTomo-HRasV12-shp53. (**B**) Test of shp53 silencing efficiency in a tree shrew liver cell line. A shRNA targeting luciferase was used as a negative control. (**C**). Kaplan-Meier curve of tree shrews infected with lentivirus. (**D**) low magnification of H&E staining of normal brain (left) and tumor (right). Tumor locates in hippocampus.

We sought to generate a tree shrew model of GBM by transducing relevant genetic mutations into neural cells *in vivo*. Recent data from the TCGA network revealed that Ras activation occur in 90% glioma patients through Receptor tyrosine kinases (RTKs), PTEN or NF-1 alterations [16, 17]. Furthermore, inactivation of TP53 is one of the most common types of genetic alterations in human GBM. To generate an accurate, disease-relevant model of glioma in tree shrew, we engineered a lentiviral vector expressing HRasV12 and a shRNA targeting tree shrew *Tp53* (Figure 2A). Due to high homology between human and tree shrew *Tp53* gene, the shRNA target sequence is 100% homology and used to silence *Tp53* in human cell in previous studies [18, 19]. The lentivirus was also tested in a tree shrew liver cell line to validate its efficiency at silencing the tree shrew p53.

As shown in Figure 2B, lentiviral infection resulted in efficient silencing of *Tp53* expression. pTomo-HRasV12-shp53 lentiviral particles were injected into the hippocampus of 1-2-year-old adult tree shrews. Tree shrews showed slight neurological symptoms such as ataxia, imbalance, or emaciation as early as four weeks after injection. The HRas-shp53 transduced tree shrew invariably developed malignant gliomas, but not developed in the control group which expresses a shRNA against luciferase and EGFP instead of H-Ras (Figure 2C). The low-magnification field of H&E staining shows the location of the tumor in the hippocampus (Figure 2D). To determine the time of tumor formation, brain was dissected weekly after injection and checked by H&E staining. Tumor formation was observed beginning at 4 weeks (Supplementary Figure 2).

### Tree shrew glioblastoma shows features typical of human glioblastoma

**Figure 3:**
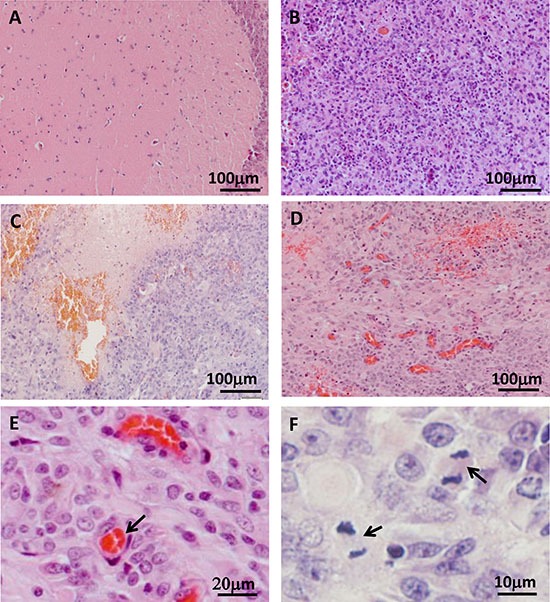
Histological examination of tree shrew brain tumors (**A**) Normal brain tissue. (**B**) High-cell-density tumor tissue. (**C**) Necrotic region. (**D**) Blood vessel hyperplasia. (**E**) Microvessel, as indicated by the arrow. (**F**) Active proliferation in the tumor. Arrows indicate cells in metaphase.

**Figure 4:**
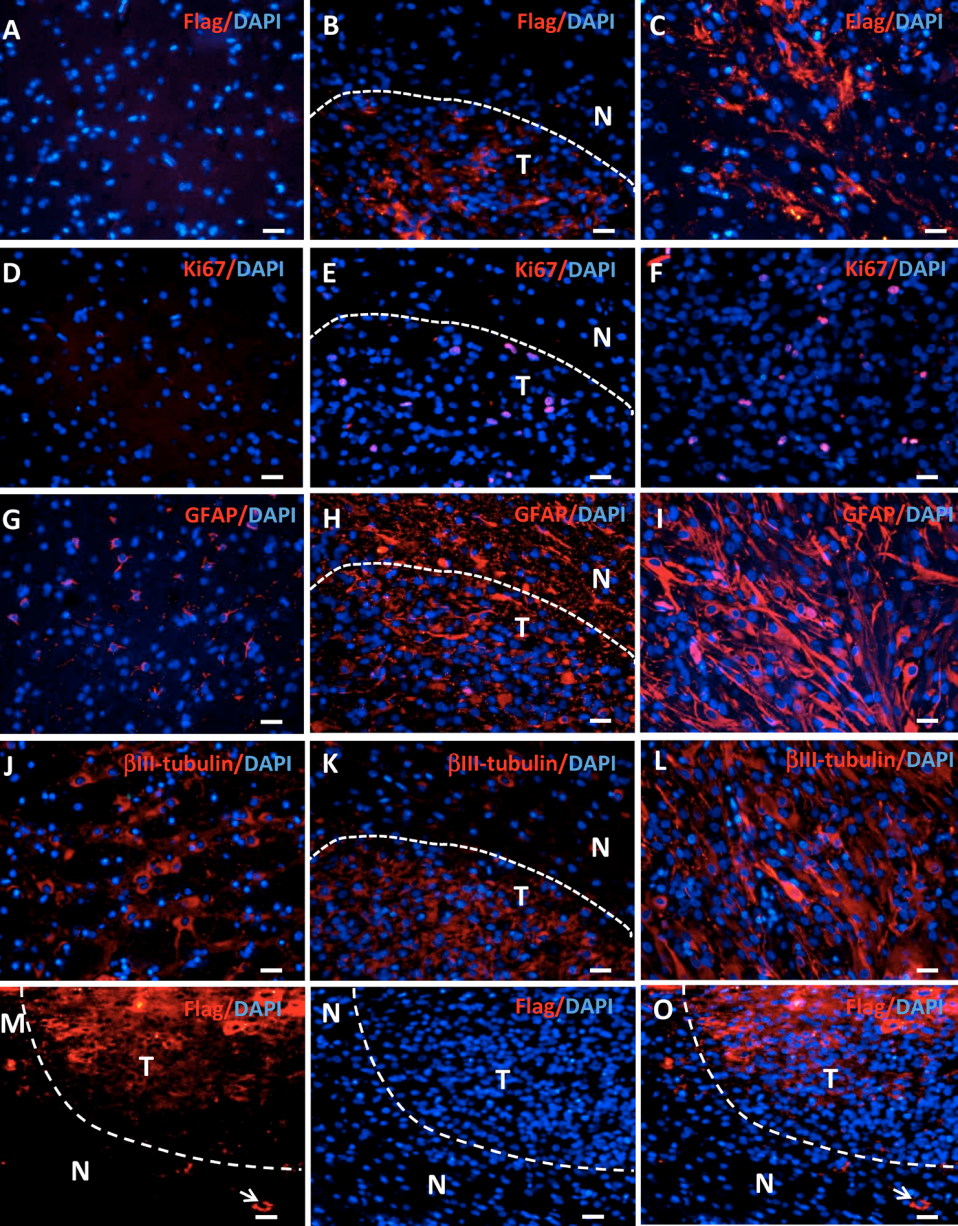
Gene expression in tree shrew brain tumors detected by immunofluorescence Staining for Flag-tagged H-Ras, Ki67, GFAP, and βIII-tubulin expression was performed in normal brains (**A**, **D**, **G**, **J**), the edge area between the tumor (T) and the normal tissue (N) (**B**, **E**, **H**, **K**), and the tumor (**C**, **F**, **I**, **L**). (**M**–**O**) Flag staining shows positive cells outside the tumor (arrows).

All tumors were excised, and their histological characteristics were analyzed. The main histological features of human glioblastoma were observed in the tree shrew model, including increased cell density (Figure 3A, 3B and Figure 4), necrosis and pseudo-palisades (Figure 3C), vascular hyperplasia (Figure 3D, 3E), and active cellular proliferation as shown by mitotic cells (Figure 3F).

H-Ras was widely expressed in the tree shrew samples, as demonstrated by Flag staining, whereas no Flag staining was evident in normal tissue (Figure 4A–4C). TP53 expression was suppressed in tumor checked by real-time PCR (Supplementary Figure 3). The Flag-positive cells outside of the tumor mass indicate the invasion of tumor cells into normal tissue, which is the one of the key features of human glioblastoma (Figure 4M–4O). The expression of genes involving in invasiveness of human glioblastoma was analyzed with RNAseq data [20]. Some genes were upregulated in tree shrew GBM tumor tissue (Supplementary Figure 4). In the tree shrew GBM, we detected increased staining for the proliferation marker Ki67 (Figure 4D–4F). The tumors also expressed the astrocyte marker GFAP (Figure 4G–4I) and the neuronal marker βIII-tubulin (Figure 4J–4L). H-Ras, Ki67 and βIII-tubulin expression showed clear borderlines; however, GFAP expression was high in both the tumor and the adjacent normal tissue. The staining patterns of these antibodies are similar to reported before and suggest the specificity to tree shrew samples. Moreover, the expression of some key factors in gliomas were detected by QPCR, such as *IDH1, TERT, EGFR, PTEN, ATRX*. Consistent with human GBM, *EGFR* was upregulated in tree shrew GBM (Supplementary Figure 5).

### The tree shrew GBM model belongs to the mesenchymal subgroup

The mouse model induced by similar lentivirus expressing HRASV12 and Tp53 shRNA was classified into mesenchymal subgroup of glioblastoma based on gene expression pattern [6]. To classify the subtype of tree shrew model, the same linear discriminant analysis (LDA) was done on tree shrew samples. Human samples including four subtype of GBM (classical, mesenchymal, neural and proneural) were treated as the training set, and 4 tree shrew samples were treated as the test set. The relative expression value of 8690 1:1 ortholog genes between tree shrew and human were used for LDA analysis (Supplementary Table 3). All four tree shrew samples clearly belonged to the mesenchymal subtype (Supplementary Table 2).

### The tree shrew GBM resembles human GBM more accurately than mouse GBM

To reveal the similarity of the global and pairwise expression patterns between tree shrew, human, and mouse, 41 mesenchymal human and 4 mouse GBM samples described previously [6] were used. By identifying 1:1:1 correlations between tree shrew, human, and mouse, 6029 orthologous genes were obtained (Supplementary Table 4).

**Figure 5:**
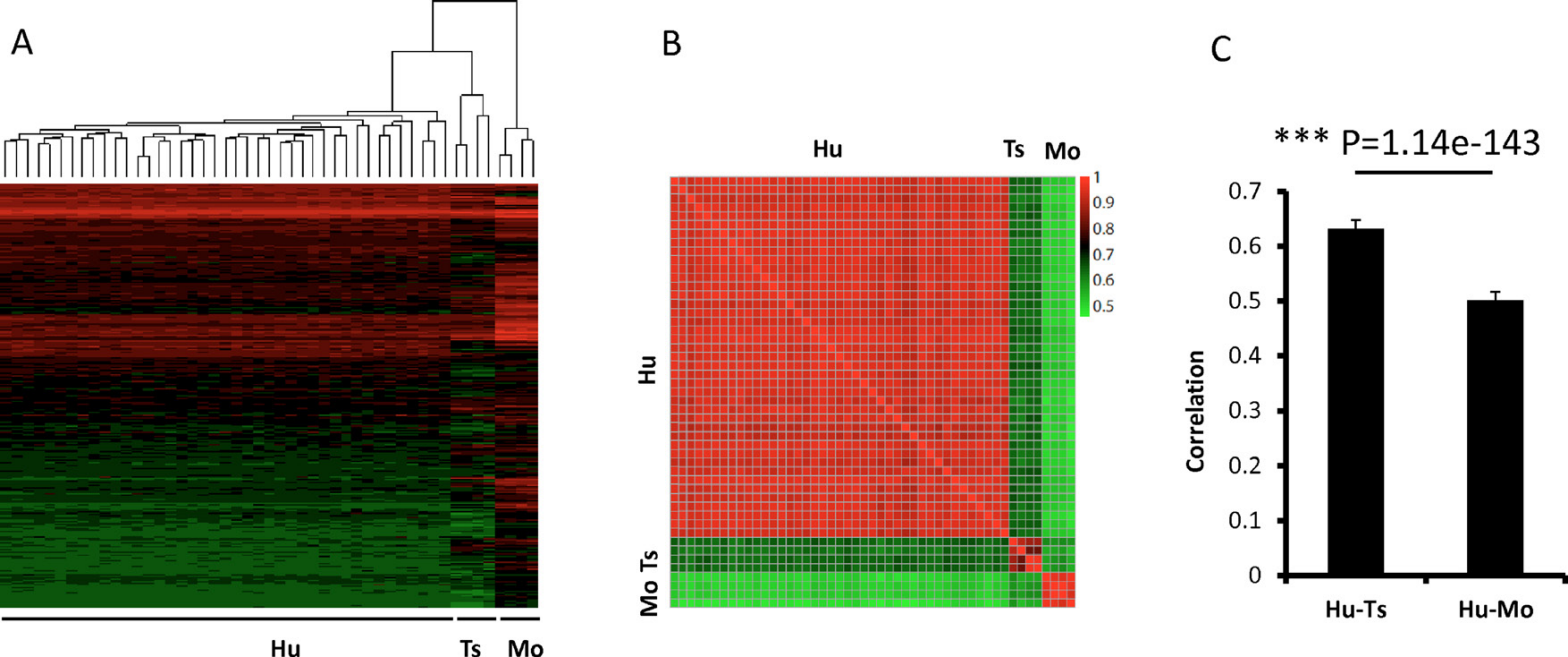
Pairwise comparisons of the gene expression profiles among tree shrew, human, and mouse glioblastomas The dendrogram (**A**) show 3 clear clusters of tree shrew, human, and mouse samples. The heatmap (**B**) illustrates the correlation of the expression profiles from the 3 species. The bar (**C**) illustrates the average correlation between the 3 species. The error bars show the standard deviations. The Red/green values represent Pearson Correlation Coefficient between the heatmap. Hu:human; Ts: tree shrew; Mo: mouse.

**Figure 6:**
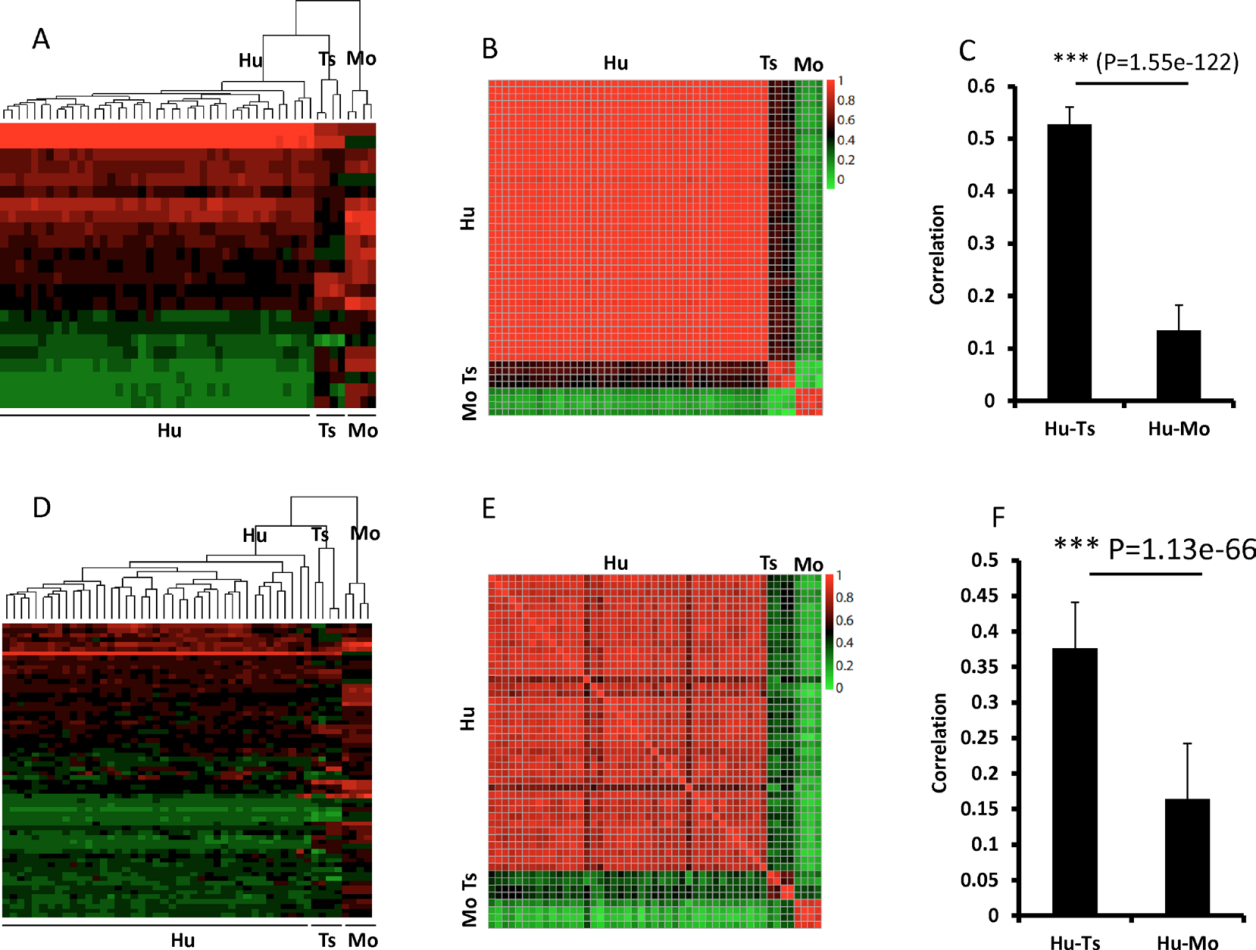
Pairwise comparisons of Ras (**A**–**C**) and Tp53 (**D**–**F**) target gene expression among tree shrew, human, and mouse. A–C, Ras target genes; D–F, Tp53 target genes. The dendrograms (A) and (D) show 3 clear clusters of tree shrew, human, and mouse. The expression heatmaps (B and E) illustrate the correlation between the 3 species. C and F illustrate the average correlation between the heatmaps in (B) and (E), respectively. The error bars show the standard deviations. The 23 genes of Ras targets are *RAC1, RHOA, RALA, RAF1, ABL1, RALGDS, RAB5A, RALBP1, TBK1, NFKB1, TIAM1, BAD, CDC42, MAPK3, ARF6, RAPGEF5, PLD2, REL, RIN1, PAK1, KSR1, PLD1*, and *PLA1A* from top to bottom of heatmap. The sixty-four genes of p53 targets are *S100A9, S100A8, ATF3, CCNB1, IER3, CDKN1A, RHOA, NFIC, E2F3, PSEN1, AKT1, MCM7, BTG2, TRIM26, PRKAB1, CHUK, RB1, ETS2, XPC, MSH2, HIPK2, CEBPA, PPP2R4, BAX, PIAS3, TBP, ING1, EZH2, BIRC5, CHGA, GDF15*, THBS1, *EGFR, IL6, STMN1, CX3CL1, PLK3, IGF2, TERT, CDC25C, E2F2, ESR1, BDKRB1, MDM2, UNC5B, FBLN1, PTHLH, ETS1, AMH, MMP13, WRN, CCNE1, CHEK2, BRCA1, CHEK1, CCNA1, EPHA2, POLD1, JAK2, FDXR, PML, PLK1, RAD51*, and *TK1* from top to bottom of heatmap. The Red/green values represent Pearson Correlation Coefficient between the heatmap. Hu:human; Ts: tree shrew; Mo: mouse.

Dendrograms of gene expression were built with the Pearson method, and the global expression patterns demonstrated 3 clear clusters of tree shrew, human, and mouse. In total, the expression pattern of tree shrew model was more closely clustered with the human pattern than the mouse pattern for all 6029 genes (Figure 5A). The same pattern was found when we analyzed Ras and p53-target genes (Figure 6A, 6D). The square matrix heatmaps and pairwise bar analyses provided independent validation to the notion that tree shrew GBM transcriptome is closer than mouse GBM to the human counterpart (Figure 5B, 5C and Figure 6B, 6C, 6E, 6F). Taken together, these findings suggest that the tree shrew model is more similar to human than mouse model at the transcriptome level both from global and single pathway pattern.

## DISCUSSION

Several cancer models have been reported in tree shrew. Spontaneous breast cancer and hepatocellular carcinoma have been found in tree shrew [21]. Induced cancer were also reported, such as hepatocellular carcinoma developed by taking advantage of its unique ability to be infected by Human hepatitis B virus, sarcomas induced by Benzopyrene, pulmonary adenomas induced by 2, 2′-dihydroxy-di-npropylnitrosamine (DHPN) [21]. However, genetic modeling of cancer in tree shrew has never been reported. Here, we propose the tree shrew as an organism for modeling of cancer through the manipulation of individual or combinatorial modules of cancer genes. Modeling cancer in tree shrew provides better outcome with relatively inexpensiveness contrast to primate model.

The mouse is the most widely used model organism. To date, there have been numerous mouse genetic engineering models of glioblastoma [22]. However, rodent models have significant differences in comparison with human cancer counterparts due to the mouse's low phylogenetic position. Analysis of genes clearly associated with human disease showed that some genes cannot be found in mouse, and some essential genes for human were not essential for mouse [7]. Because the function of essential genes should be more conserved than that of nonessential genes, so the general differences between human and mouse functional genomics are even more significant. Furthermore, there is a significant difference between human and mouse immune system, including immune system structure, innate and adaptive immunity [8]. These differences lead to the failure of many research results performed in mice to be applied in human, especially for drug development, which needs to balance many factors such as pharmacodynamics, pharmacokinetics, and toxicology. Only 10.4% of drugs tested in mice produced the expected clinical response [23, 24].

Although there are some disadvantages, including lack of inbred line, being more difficult to maintain and manage than mouse, and being easily to be frightened, the tree shrew has been proposed as a valuable animal model for several decades due to its small body size, short period to sexual maturation (4–6 months), low cost of maintenance compared to primate and potential as an alternative to primates [10, 11]. The evolutionary status of the tree shrew has been debated for a long time. Tree shrew was considered to be more closely related to Scandentia, Dermoptera or Primates based on different evidences from the mitochondrial genome, molecular cytogenetic data and multiple nuclear genes [25, 26]. This phylogenic conflict has been a huge obstacle for the use of tree shrews in biomedical research. We completed genome sequencing of the Chinese tree shrew which provides strongest evidence for its evolutionally closest relationship to primates and therefore should provide advantages over the mouse in many biomedical research fields [10]. Drug-targeting analysis has also indicated the advantages of tree shrew models over mouse models [27]. In particular, the tree shrew is more similar to primates with respect to the nervous system, with a high brain-to-body mass ratio, a well-developed neocortex, versatile limbs, and vision and hearing degeneration [10, 11, 28, 29].

Recently, extensive analysis of glioblastoma genomics showed that the receptor tyrosine kinase pathway (RTK pathway) is activated in approximately 90% of GBM [17, 30]. Ras is a critical node in the RTK pathway and activated in most GBM samples. Other frequent genetic alterations include mutations in *Tp53* (approximately 30%) and *CDKN2A* (approximately 60%), which are exclusive to each other in human glioblastoma [17]. Therefore, Ras activation and *Tp53* alteration represent typical genetic changes in glioblastoma. Previous research has demonstrated that a mouse model of glioblastoma can be induced with high efficiency by infecting hippocampal cells with lentivirus for overexpression of constitutively active HRasV12 and *Tp53* silencing [6, 31]. This model mimics the main features of the mesenchymal subtype of human glioblastoma [6]. In this study, similar lentiviral particle, except for the replacement of shRNA against mouse *Tp53* by shRNA targeting tree shrew *Tp53*, successfully induced glioblastoma in tree shrew. The induced tree shrew glioblastoma shared more similarities to human samples than mouse model.

The homology of most genes to human orthologous is higher in tree shrew than in mouse. This is especially true for crucial cancer genes such as *Tp53* and *CDKN2A* that harbor frequent genetic alterations (mutations and/or copy number changes) in human GBM. The tree shrew Tp53 protein shares much higher homology with the human protein and has more conserved post-translational modification sites than mouse Tp53 (Figure 1). This evidence encouraged us to generate a glioblastoma model in tree shrew to better mimic human disease. Indeed, the tree shrew glioblastoma model showed an expression profile that was more similar to the human disease than mouse model especially with respect to the Ras and *Tp53* pathway.

One of the most important uses of animal models is for drug discovery. The overall analysis of metabolism, target and side effect-related genes suggests that the tree shrew will be more advantageous than the mouse model for drug screening programs [10, 32]. In particular, the most important genes for drug metabolism, the cytochrome P450 superfamily, exhibit large differences between human and mouse, while similar gene structure and homologous sequence is found in tree shrew [10]. Global analysis of drug targeting in tree shrew has already shown significant advantages in comparison to mouse [27]. Thus, the tree shrew model reported here provides a more accurate reproduction of human brain tumors that will be of better use for GBM research and drug discovery.

## MATERIALS AND METHODS

### Tree shrews

Adult male Chinese tree shrews (*Tupaia belangeri chinensis*, *N* = 8) weighing 120−150 g were obtained from a breeding colony at the Kunming Primate Research Center, Kunming Institute of Zoology, CAS. All animals were provided free access to food and water. All animal care and experimental protocols were approved by the Animal Care and Use Committee of Kunming Institute of Zoology, Chinese Academy of Sciences, P. R. China.

### Lentivirus production

The pTomo H-RasV12 lentiviral vector was a gift from the Verma lab [5]. The pTomo-H-RasV12-shp53 vector was constructed by inserting an H1 promoter-driven shRNA against tree shrew *Tp53* into the ClaI site of the pTomo-H-RasV12 vector. The shRNA sequence targeting tree shrew *Tp53* is CACCATCCACTACAACTACAT, and the silencing efficiency was detected by real-time PCR with *Tp53* real-time primers (5′-GCACCACCATCCACTACAAC-3′ and 5′-TCTGTGCGTCGGTCTCTTCCA-3′) and 18S RNA primers (5′-TTCGGAACTGAGGCCATGAT-3′ and 5′-TTTCGCTCTGGTCCGTCTTG-3′) as reference gene. To prepare lentiviral particles, the lentiviral vector was co-transfected with pCMV-dR8.1 and pCMV-MD2.G plasmids into human embryonic kidney (HEK) 293T cells by calcium phosphate transfection. The supernatant containing the lentiviral particles was harvested twice at 2 days and 3 days after transfection. The supernatant was then concentrated via super centrifugation for 2 hours. The pellet was suspended in PBS containing 0.1% BSA, and the aliquots were stored at −80°C. The titer was determined by real-time PCR.

### Intracranial injection

The tree shrews were anesthetized with ketamine and fixed onto a stereotaxic apparatus. Lidocaine hydrochloride was administered locally to further alleviate pain after the head skin was shaved and sterilized. Then, 4 μl lentivirus (3 × 10^11^/ml titered by real-time PCR) was injected into the hippocampus with pre-identified parameters of 6 mm in depth from the skull and 7 mm lateral and 14.2 mm posterior to bregma. The needle was kept in place for five minutes after the injection was completed over the course of 10 minutes. All tree shrews were given good care and recovered well.

### Tissue preparation and HE staining

Fresh tissue was immediately frozen by liquid nitrogen and stored at −80°C. To collect fixed tissue, tree shrews were anesthetized and perfused with 4% paraformaldehyde through the heart and vascular system. The brain and the tumor were surgically removed and fixed in 10% neutral-buffered formalin for 72 hours. Standard procedures were then used to produce paraffin-embedded sections for hematoxylin and eosin (HE) staining and immunofluorescence staining.

### Immunofluorescence

Sections were deparaffinized in xylene and rehydrated in a graded series of alcohol. Antigen retrieval was performed for 5 min at 125°C in citrate buffer (pH 6.0). Following standard procedures, the slides were incubated with 3% H_2_O_2_ to inactivate endogenous peroxidase, blocked for more than one hour in 10% serum in PBS with 0.1% Triton X-100, and then incubated with primary antibodies and Cy3-labeled secondary antibodies. The nuclei were stained with DAPI. The antibodies used include Ki67 (Vector Labs, Cat# vp-k452, 1:200), GFAP (Dako, Cat# Z0334, 1:200), βIII-tubulin (Promega, Cat# G7121, 1:1000) and Flag (Sigma, Cat# F2555, 1:125).

### Tree shrew tumor RNAseq and transcriptome analysis

Total RNA was separately extracted from 4 tree shrew tumor samples. The RNA sequencing libraries were constructed using the Illumina mRNA-Seq Prep Kit. All the libraries were sequenced on the Illumina HiSeq 2000 platform in paired-end form with 101bp. The total ~5 Gb raw sequence data was obtained for each sample. The raw sequence reads have been deposited in http://gsa.big.ac.cn/index.jsp (accession no. PRJCA000125). Low-quality reads were filtered out using the Perl script IlluQC.pl from GSQCToolkitv2.3.3 with the parameters –pe-cutOffReadLen4HQ 70–cutOffQualScore 20 N 5. The clean reads were mapped to the tree shrew genome (tch_ref_TupChi_1.0_chrUn) using tophat-2.1.0 and read-mismatches 2, and the expected fragments per kilobase of transcript per million fragments (FPKM) of the genes were calculated using express cufflinks2.02, --max-multiread-fraction 0.75.

### Classification of tree shrew tumor samples

Seventy human GBM samples, which were used in our previous work to classify mouse glioblastoma [6], were also used in this study. To compare the microarray data from the human samples with the RNA-seq data from the tree shrew samples, the tree shrew and human GBM expression data were normalized using relative values as follows: Z-score(i) = (raw(i)-mean(x))/sd(x) [33], where x is all of the expression data in the same sample, i is the ith gene expression data, raw(i) is the raw expression of the ith gene, mean(x) is the average value of x, and sd(x) is the standard deviation of x. Positive Z-score values denote higher expression than the average gene expression. In contrast, negative values denote lower expression than the average.

Next, to classify the tree shrew samples, we applied the LDA implemented in the R package MASS as previously described [6]. The 70 human and 4 tree shrew GBM samples were treated as the training and test sets, respectively.

### Glioma mRNA expression pattern between tree shrew, human, and mouse

The expression data of 41 human mesenchymal and mouse GBM samples induced by lentiviral overexpression of H-Ras V12 and *Tp53* silencing are the same as in our previous paper [6]. Raw data were log-transformed according to the method described above, and then the expression data from 3 species was normalized using relative values. The heatmap.2 program implemented in the R package plots was applied to illustrate differences in the expression patterns of the 3 species.

The Ras target genes were derived from the Ras signaling pathway (KEGG no: hsa04014, 2014.09.19), and 23 of 24 target genes were 1:1:1 orthologs between human, tree shrew and mouse. The Tp53 target genes were from the database TRED (https://cb.utdallas.edu/cgi-bin/TRED/) [34], and 64 of 187 verified target genes were 1:1:1 orthologs between human, tree shrew and mouse.

## SUPPLEMENTARY MATERIALS FIGURES AND TABLES






