# Supplementary material for: A tree shrew glioblastoma model recapitulates features of human glioblastoma
Source: Oncotarget. 2017 Feb 9;8(11):17897–907. doi: 10.18632/oncotarget.15225 (PMC5392295; doi:10.18632/oncotarget.15225)
Supplement: Supplementary file 1 [file oncotarget-08-17897-s001.pdf]

A tree shrew glioblastoma model recapitulates features of human glioblastoma

Supplementary Materials

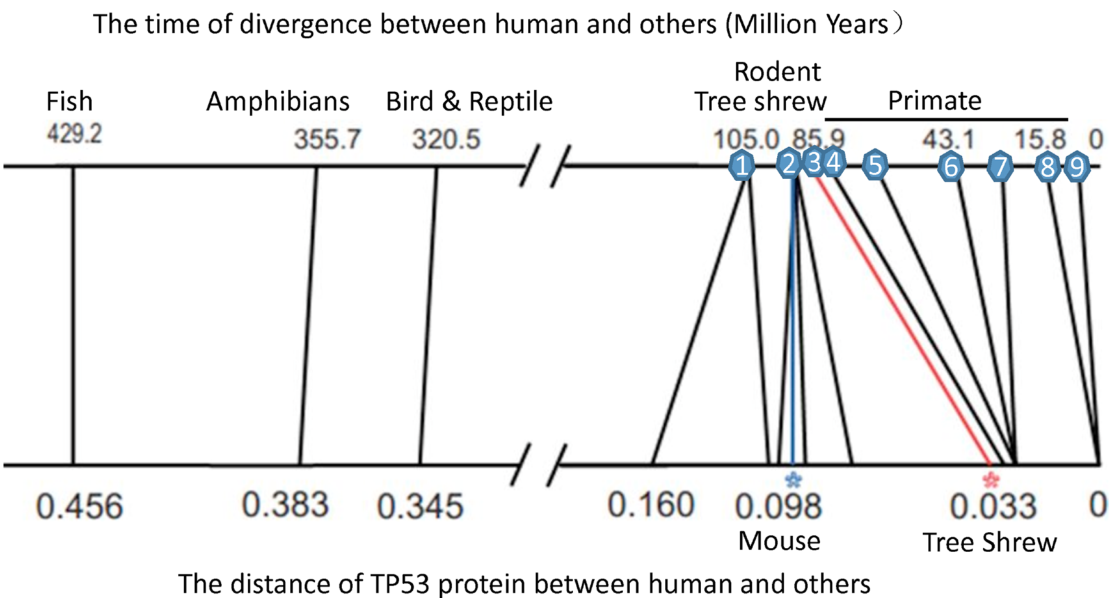

Supplementary Figure 1: Evolutionary analysis of the amino acid sequences of Tp53 from 87 species. The upper line denotes the divergence time point (million years). The bottom line is the distance of the Tp53 proteins of other species from the human protein.

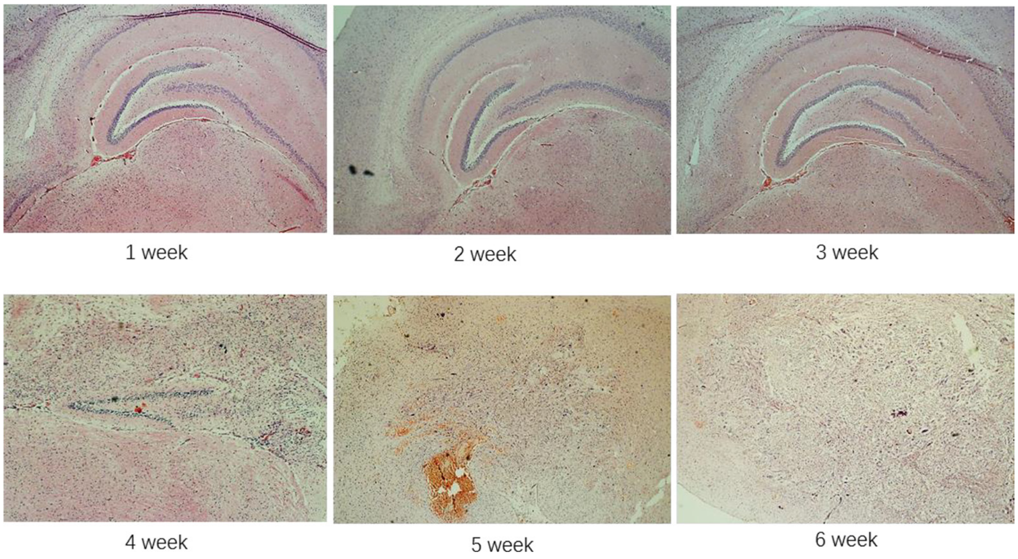

Supplementary Figure 2: Pathological detection of tree shrew weekly after injection with lentiviral particles. 2 tree shrews were dissected at each time point.

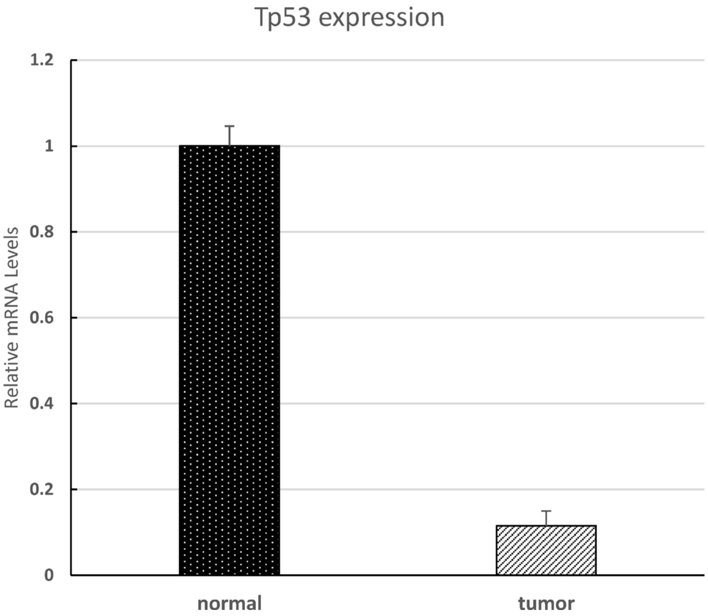

Supplementary Figure 3: The expression of TP53 in tree shrew GBM.

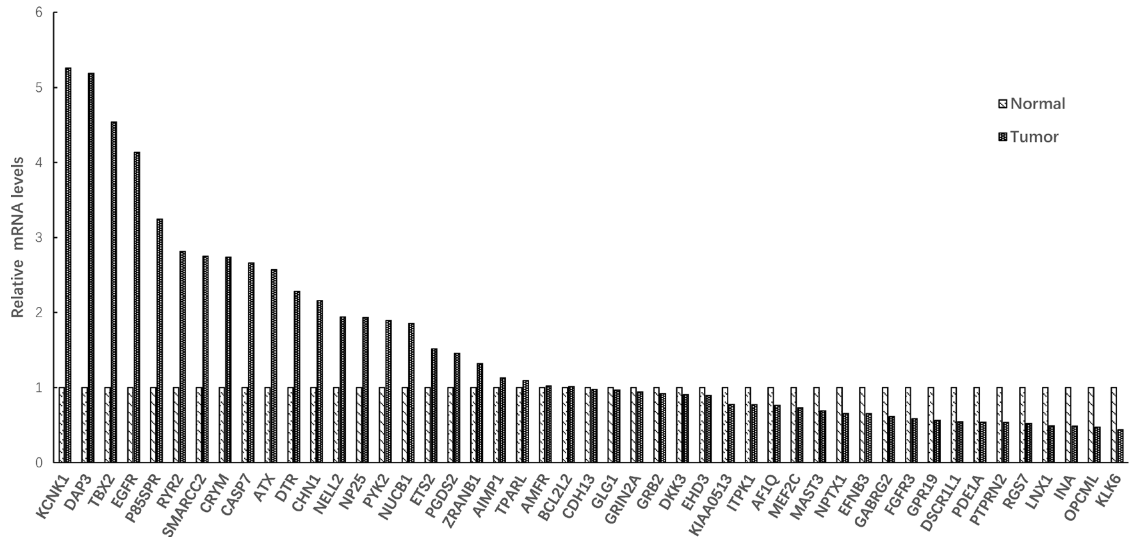

Supplementary Figure 4: The expression of genes upregulated in human GBM invasive cells in tree shrew GBM.

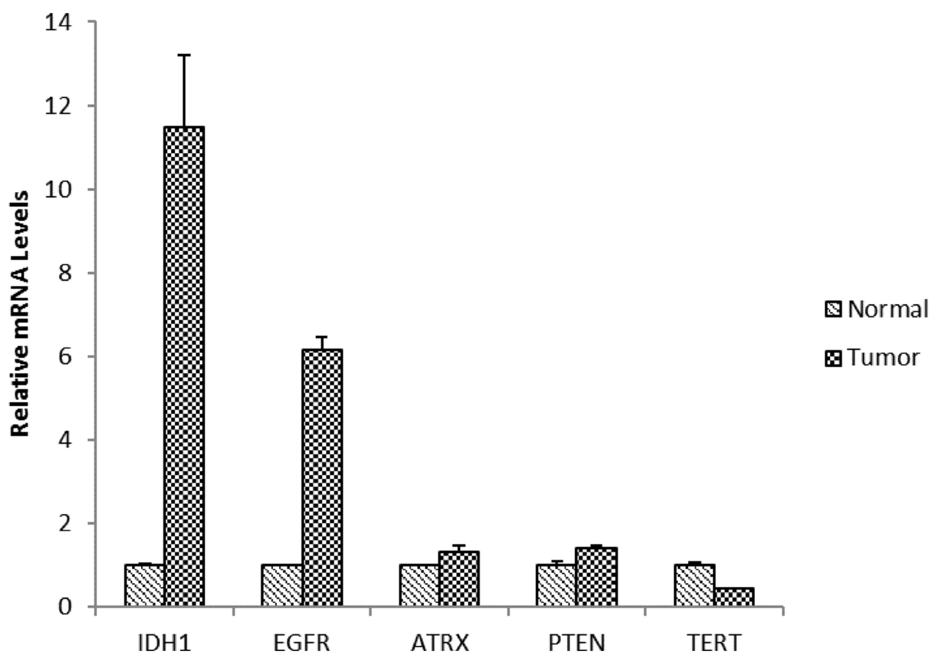

Supplementary Figure 5: The expression status of some key factors of gliomas in tree shrew GBM, such as IDH1, TERT, EGFR, PTEN, ATRX, et al.

Supplementary Table 1: Summary of post-translational modification site differences between mouse and tree shrew

Site	Modification	Modifying enzyme	Function	Implication of Cancer
S33	phosphorylation	CDK5[1], CDK7[2], CDK9[3], CAK[4], P38 (MAPK)[5]	Interaction with CBP/p300 proteins[3], Interaction with Pin1 protein[13], P53 stabilizatio [5]	breast cancer[13], lung carcinoma[15]
P36	phosphorylation			
S37	phosphorylation	hCHK1[4], ATR[4], DNAPK[4], PRAK[6]	Interaction with CBP/p300 proteins[3], Promoting apoptosis[5], Disruption of the MDM2-P53 complex[5]	colon carcinoma[15]
S46	phosphorylation	CDK5[1], HIPK2[5], P38 (MAPK)[5], PKCδ[7], DYRK2[8]	Interaction with Pin1 protein[13], Promoting cell migration[13] Facilitating of P53 acetylation[5], Inducing apoptosis[8]	oral squamous cell carcinoma[16], breast cancer[13],
T55	phosphorylation	TAF1[9], GRK5[10], ERK2[5]	P53 nuclear export[3], Promoting P53 degradation[9], Activating P53[5],	breast cancer[17]
T81	phosphorylation	JNK[5]	Facilitating P53 transcriptional activity[14] Stabilizing of P53[14],	breast cancer[13] bladder cancer[18]
S149	Phosphorylation	CSN-associated kinase complex[11]	Promoting P53 degradation[3]	breast cancer[19], osteosarcoma[19]
S149	O-glcNAcylation	O-GlcNac transferase[11]	Stabilizing of P53[11]	
T150	phosphorylation	CSN-associated kinase complex[11]	Promoting P53 degradation[3]	breast cancer[19], osteosarcoma[19],
T155	phosphorylation	CSN-associated kinase complex[11]	Promoting P53 degradation[3]	small cell lung cancer[20], hepatocellular carcinoma[21], breast cancer[19], osteosarcoma[19],
K357	mono-ubiquitination	MSL2 [12]	P53 nuclear export[12]	

REFERENCES

1. Lee JH, Kim HS, Lee SJ, Kim KT. Stabilization and activation of p53 induced by Cdk5 contributes to neuronal cell death. J Cell Sci. 2007; 120:2259–2271.
2. Ko LJ, Shieh SY, Chen XB, Jayaraman L, Tamai K, Taya Y, Prives C, Pan ZQ. p53 is phosphorylated by CDK7-cyclin H in a p36(MAT1)-dependent manner. Molecular and cellular biology. 1997; 17:7220–7229.
3. Ji XN, Huang Q, Yu L, Nussinov R, Ma BY. Bioinformatics Study of Cancer-Related Mutations within p53 Phosphorylation Site Motifs. International journal of molecular sciences. 2014; 15:13275–13298.
4. Shieh SY, Ahn J, Tamai K, Taya Y, Prives C. The human homologs of checkpoint kinases Chk1 and Cds1 (Chk2) phosphorylate p53 at multiple DNA damage-inducible sites (vol 14, pg 289, 2000). Gene Dev. 2000; 14:750–750.
5. Bode AM, Dong ZG. Post-translational modification of p53 in tumorigenesis. Nat Rev Cancer. 2004; 4:793–805.
6. Sun PQ, Yoshizuka N, New L, Moser BA, Li YL, Liao R, Xie CC, Chen JM, Deng QD, Yamout M, Dong MQ, Frangou CG, Yates JR, et al. PRAK is essential for ras-induced senescence and tumor suppression. Cell. 2007; 128:295–308.
7. Yoshida K, Liu HS, Miki Y. Protein kinase C delta regulates Ser(46) phosphorylation of p53 tumor suppressor in the apoptotic response to DNA damage. Journal of Biological Chemistry. 2006; 281:5734–5740.
8. Taira N, Nihira K, Yamaguchi T, Miki Y, Yoshida K. DYRK2 is targeted to the nucleus and controls p53 via Ser46 phosphorylation in the apoptotic response to DNA damage. Mol Cell. 2007; 25:725–738.
9. Li HH, Li AG, Sheppard HM, Liu X. Phosphorylation on Thr-55 by TAF1 mediates degradation of p53:a role for TAF1 in cell G1 progression. Mol Cell. 2004; 13:867–878.
10. Chen X, Zhu H, Yuan M, Fu J, Zhou Y, Ma L. G-protein-coupled receptor kinase 5 phosphorylates p53 and inhibits DNA damage-induced apoptosis. The Journal of biological chemistry. 2010; 285:12823–12830.
11. Kruse JP, Gu W. SnapShot:p53 posttranslational modifications. Cell. 2008; 133:930–930 e931.
12. Lee JT, Gu W. The multiple levels of regulation by p53 ubiquitination. Cell death and differentiation. 2010; 17:86–92.
13. Girardini JE, Napoli M, Piazza S, Rustighi A, Marotta C, Radaelli E, Capaci V, Jordan L, Quinlan P, Thompson A, Mano M, Rosato A, Crook T , et al. A Pin1/mutant p53 axis promotes aggressiveness in breast cancer. Cancer cell. 2011; 20:79–91.
14. Buschmann T, Potapova O, Bar-Shira A, Ivanov VN, Fuchs SY, Henderson S, Fried VA, Minamoto T, Alarcon-Vargas D, Pincus MR, Gaarde WA, Holbrook NJ, Shiloh Y , et al. Jun NH2-terminal kinase phosphorylation of p53 on Thr-81 is important for p53 stabilization and transcriptional activities in response to stress. Molecular and cellular biology. 2001; 21:2743–2754.
15. Sakaguchi K, Herrera JE, Saito S, Miki T, Bustin M, Vassilev A, Anderson CW, Appella E. DNA damage activates p53 through a phosphorylation-acetylation cascade. Gene Dev. 1998; 12:2831–2841.
16. Dai C, Gu W. p53 post-translational modification:deregulated in tumorigenesis. Trends Mol Med. 2010; 16:528–536.
17. Levy CB, Stumbo AC, Bom APDA, Portari EA, Carneiro Y, Silva JL, De Moura-Gallo CV. Co-localization of mutant p53 and amyloid-like protein aggregates in breast tumors. Int J Biochem Cell B. 2011; 43:60–64.
18. Yoshimura I, Kudoh J, Saito S, Tazaki H, Shimizu N. P53 Gene Mutation in Recurrent Superficial Bladder-Cancer. J Urology. 1995; 153:1711–1715.
19. Bourdon JC, Fernandes K, Murray-Zmijewski F, Liu G, Diot A, Xirodimas DP, Saville MK, Lane DP. p53 isoforms can regulate p53 transcriptional activity. Gene Dev. 2005; 19:2122–2137.
20. Kanashiro CA, Schally AV, Groot K, Armatis P, Bernardino ALF, Varga JL. Inhibition of mutant p53 expression and growth of DMS-1 53 small cell lung carcinoma growth hormone-releasing by antagonists of hormone and bombesin. Proceedings of the National Academy of Sciences of the United States of America. 2003; 100:15836–15841.
21. Lim SO, Kim H, Jung G. p53 inhibits tumor cell invasion via the degradation of snail protein in hepatocellular carcinoma. Febs Letters. 2010; 584:2231–2236.

Supplementary Table 2: LDA ananlysis of tree shrew glioblastoma

	Predicted group			
**Tumor sample**	classical	mesenchymal	neural	proneural
shrew_tree1	0.13	0.51	0.35	0.01
shrew_tree2	0.34	0.51	0.15	0
shrew_tree3	0	0.99	0	0
shrew_tree4	0	0.98	0.02	0

Supplementary Table 3: The relative expression of human-Tree shrew 1:1 ortholog genes. See Supplementary_Table_3

Supplementary Table 4: The relative expression of human-Tree shrew-mouse 1:1:1 ortholog genes. See Supplementary_Table_4
